# How a Star Is Weighed

**Published:** 1887-06

**Authors:** Paul A. Towne


### HOW A STAR IS WEIGHED.

BY PROFESSOR PAUL A. TOWNE.

The power we have of weighing a star is, without doubt, one of the most surprising results of the advancement of the sciences, that one indeed which persons unacquainted with the principles of celestial mechanics most hesitate to accept. To weigh a star is a fact more extraordinary, again, than to measure the distance of one; and certainly, neither Copernicus, nor Galileo, nor Kepler, nor Newton could have imagined that the day would come when their successors would be able, by the application of their immortal discoveries, to determine the mass of a star moving in the depths of celestial space. Let us attempt to give an idea of the method employed in acquiring a knowledge of the magnitude and masses of stars.

The mass of a star is calculated by the energy of the action that it throws around it. If the earth were ten times heavier than it is, still preserving the same volume, it would draw bodies toward its surface ten times more forcibly than it now does, and an object which now falls a given number of feet in the first second of time, would then drop ten times that number of feet in that second. Again, if the earth, still preserving its volume, had the mass of the sun, it would attract bodies with an energy increased 324,000 times, and an object which now weighs one pound would then weigh 324,000 pounds; a man of the mean weight of 160 pounds would weigh 51,000,000 of them! We measure the weight of a star by the intensity of the attraction to its surface. Reduced to its simple expression, in its application to the fall of bodies, this attraction would be hard to verify; but we can determine it by the velocity of a satellite gravitating around a star whose mass we wish to know.

For example, the attraction of the earth has the power of curving the straight line which would be followed by the moon in space if this attraction did not exist, and it bends the line by its attraction in such a way that the moon runs round the circumference of a circle in twenty-seven days, seven hours and forty-three minutes. If the mass, or the energy of the earth should increase, the velocity of the moon in its orbit would also be augmented; if the mass should be diminished, the contrary effect on the moon's orbit would be produced. Attraction varies in the direct ratio of the masses. The velocity of the moon around the earth comes from this same force of the earth. The earth is the hand which causes the moon to turn in the sling. If the earth had more force, more energy than it really has, it would cause the moon to turn more swiftly and vice-versa. If the sun should increase in weight the earth and the other planets would turn more rapidly around it, and years would decrease in length. If the mass of the sun should decrease, the contrary results would take place. By comparing the action of the sun on the earth with the action of the earth on the moon, we have found that the sun is 324,000 times more energetic, more powerful, more heavy than the earth.

If then we had in space a celestial couple of which the mutual distance of the two components were equal to that which separates the earth from the sun, or 91,500,000 miles, the examination of the duration of its revolution would give us immediately the mass of the sun. Mathematically speaking, if a couple of celestial bodies turning around their common centre of gravity employs a certain time, T, to accomplish its revolution, while another pair, whose components are the same distance from each other, employs another time, D, to accomplish its revolution, the mass of the first pair is to the mass of the second in the inverse ratio of the square of the times; that is, as $D^2$. is to $T^2$. If the distance is not the same, it is necessary first to reduce it to this quality, in taking account of the law which governs distances:—"The squares of the periodic times are to each other as the cubes of the distances."

In this way Camille Flammarion, the eminent French astronomer, has been able to calculate the mass of the stellar system of the double star (Rho) Ophiuchus. By a combination of all the observations, he found that the period is 95 years and 283 days. The parallax of the star, being 0 ... 168, corresponds to a distance from the earth equal to 1,400,000 times that of the sun. And this immense distance, the radius of the terrestrial orbit being reduced to the preceding angle, the semi-major axis, of 4 .. .88, represents 2,687,000,000 of miles. This is a little less than the distance of Neptune from the sun. A planet situated at this distance from the sun would accomplish its revolution in 156.55 years. The ratio then is the square of 156.55 to the square of 92.77 or as 2.85 to 1, from which it is concluded that the mass of the system of Ophiuchus is almost three times greater than that of the sun and Neptune combined, or (Neptune having only a small relative mass) three times greater than that of the Sun alone. Thus a star has been found, hardly visible to the naked eye, which weighs 900,000 times more than the earth.

It may here be remarked that the orbital motion of the little star around the large one is about 519,000 miles per day, and that these twin suns travel together across immensity with a velocity whose minimum is 615,000,000 of miles per year. And these are among the heavenly bodies which are still called fixed stars.

Calculations made on other stars lead to similar results, presenting to us these celestial torches as gigantic and ponderous stars, that the enormous distance which separates us from them reduces to simple mathematical points. The star nearest to us, Alpha of Centaurus, has a parallax of 0 .. .91 and, therefore, its distance from the earth is about twenty trillions of miles. If we adopt 15".5 for the mean value of the angle comprised between the two components, their mutual distance is found to be about 1,675,000,000 of miles. This is less than the distance of Uranus from the sun. But its period appears to be about 77 years. We infer, then, that it weighs a little less than our sun, and that representing the mass by 10, that of the sun would be represented by 12. But its volume should be larger (that is, of the two united suns) for its intrinsic light is about three times superior to that of our sun. If quantity of light is regarded as a criterion of the surface of emission, the diameter exceeds that of the sun in the ratio of 17 to 10.

The period of Eta of Cassiopeia, which at first was valued at 700 years, is now fixed at 176, and it is probable that this figure does not vary much from the truth. Allowing the parallax of 0''.154 which gives a distance of 132 trillions of miles and a semi-major axis of 10''.68, the mass of this system exceeds that of the sun ten times. It may be concluded, therefore, that double stars are veritable suns, immense and mighty, governing, in the parts of space lighted by their splendor, systems different from that of which we form a part. We infer that the sky is not a gloomy desert; all its regions may be peopled like those in which the earth happens to be located; obscurity, silence, death, once regarded as reigning in far-off distances, have given place to light, motion, to life; thousands and millions of suns pour in vast waves into space the energy, the heat and the diverse undulations which emanate from their fires. All their movements follow each other, interfere, contend or harmonize in the maintenance and incessant development of different organizations of universal life. How different the universe from that contemplated previous to the developments of modern astronomy! Suns succeed suns, worlds succeed worlds, universes succeed universes. Tremendous movements carry all the starry systems across the endless regions of immensity, and everywhere, even beyond the farthest limits to which the imagination has carried its weary flight—everywhere, divine creation shows itself in infinite variety, and our microscopic planet is one of its minutest provinces.—*The Earth.*

